# Polyvinyl Alcohol/Graphene Oxide Conductive Hydrogels via the Synergy of Freezing and Salting Out for Strain Sensors

**DOI:** 10.3390/s22083015

**Published:** 2022-04-14

**Authors:** Jingjiang Wei, Rongjie Wang, Fei Pan, Zhengyi Fu

**Affiliations:** 1 State Key Laboratory of Advanced Technology for Materials Synthesis and Processing, Wuhan University of Technology, Wuhan 430070, China; sirk@whut.edu.cn (J.W.); rjwang@whut.edu.cn (R.W.); 2 Laboratory for Biointerfaces, Swiss Federal Laboratories for Materials Science and Technology (Empa), Lerchenfeldstrasse 5, 9014 St. Gallen, Switzerland; fepan@ethz.ch

**Keywords:** polyvinyl alcohol, graphene oxide, conductive hydrogels, strain sensors, salting out

## Abstract

Hydrogels of flexibility, strength, and conductivity have demonstrated broad applications in wearable electronics and soft robotics. However, it is still a challenge to fabricate conductive hydrogels with high strength massively and economically. Herein, a simple strategy is proposed to design a strong ionically conductive hydrogel. This ion-conducting hydrogel was obtained under the synergistic action by salting out the frozen mixture of polyvinyl alcohol (PVA) and graphene oxide (GO) using a high concentration of sodium chloride solution. The developed hydrogel containing only 5 wt% PVA manifests good tensile stress (65 kPa) and elongation (180%). Meanwhile, the PVA matrix doped with a small amount of GO formed uniformly porous ion channels after salting out, endowed the PVA/GO hydrogel with excellent ionic conductivity (up to 3.38 S m^−1^). Therefore, the fabricated PVA/GO hydrogel, anticipated for a strain sensor, exhibits good sensitivity (Gauge factor = 2.05 at 100% strain), satisfying working stability (stably cycled for 10 min), and excellent recognition ability. This facile method to prepare conductive hydrogels displays translational potential in flexible electronics for engineering applications.

## 1. Introduction

The wide application of emerging flexible electronic products in smart wearable devices [1,2,3], biomedicine [4,5], soft robotics [6,7], and other fields [8] has drawn more and more attention. Traditional flexible electronics are fabricated by assembling stretchable electrode arrays by integrating methods such as microfabrication and transfer printing [9,10]. Another approach is the utilization of conductive materials, such as conducting polymers, liquid metals, and nanoelectrodes to directly construct stretchable devices [11,12,13]. Despite the success of some products, most flexible electronic devices still rely on inorganic electrode materials [14]. Due to the soft and bendable properties of human skin, wearable electronic devices are subject to various deformations, such as stretching and squeezing. Biological tissue is soft and contains a lot of water, which many bioelectronic devices are physically or mechanically unable to match [15,16]. Therefore, developing flexible and stretchable conductive materials is of practical significance for bioelectronics. In terms of applications, stretchable conductive materials also need to display good mechanical properties to enable long-term operational stability [17]. In addition, the biocompatibility of such materials cannot be ignored as they are in contact with human skin [18,19,20]. These various functions cannot be fulfilled by the existing electronic conductors. Therefore, fabricating such materials identical to human tissue remains a challenge.

Due to the unique 3D polymer network structure of hydrogels, they exhibit similar water-richness, mechanical properties, and biocompatibility to human tissues [21,22,23]. Thus, they are considered ideal materials for human implants and wearable devices [21,24,25]. However, conventional hydrogels usually lack electrical conductivity, limiting their applications in human–machine interaction [26]. Unlike traditional hydrogels, conducting polymer hydrogels have a tissue-like advantage while exhibiting electrical conductivity, which distinguishes them as the ideal materials for emerging bioelectronic devices [27,28,29]. In particular, the 3D polymer network inside the ion-conducting hydrogel makes it solid and provides a channel for ion transport, manifesting excellent ionic conductivity [30]. Graphene oxide (GO) is obtained by oxidizing graphite, and its oxygen functional groups mainly exist in the form of hydroxyl and epoxy groups, so it exhibits strong hydrophilicity [31,32]. PVA hydrogels have excellent mechanical strength and water retention ability and can be easily prepared into ionic conductive hydrogels. Therefore, the numerous hydrophilic functional groups in GO can facilitate its combination with PVA to form a more complex network structure channel, which is more conducive to the ion transport inside the hydrogel. Due to the excellent ion transport efficiency, ion-conducting hydrogels have become ideal candidates for strain sensors [33,34,35]. Nevertheless, the poor strain capacity and mechanical strength of hydrogels severely restrain their further applications [36]. However, it is a challenge to fabricate hydrogels with excellent mechanical strength and high electrical conductivity simultaneously, as these two properties are usually mutually exclusive [34]. A highly dense cross-linked network can endow hydrogels with high mechanical properties; however, this inevitably inhibits the mobility of polymer chains and reduces their electrical conductivity [34]. Recently, the conductivity and toughness of hydrogels have been increased by adding nanoparticles or forming dual networks [37,38,39]. However, the performance improvements of these toughened hydrogels are still limited.

To simultaneously improve the mechanical properties and electrical conductivity of hydrogels, here, a simple method under the synergistic action by combining freezing and salting out was employed to prepare polyvinyl alcohol (PVA) hydrogels. This hydrogel was prepared by ultrasonically degassing an aqueous PVA solution in a mold, freezing it, and then soaking it in an aqueous NaCl solution overnight. The whole preparation process is simple and suitable for massive production. Meanwhile, introducing a small amount of GO (0.15 wt%) can form a more complex PVA/GO network structure through hydrogen bonding with the PVA chain. Hence the PVA/GO hydrogel containing only 5 wt% PVA exhibits excellent mechanical properties (tensile stress up to 65 kPa) and ionic conductivity (up to 3.38 S m^−1^). Thus, an encapsulation of such ion-conducting hydrogel in stretchable insulating tapes can lead to the successful development of a PVA/GO strain sensor. The strain sensor can be attached to a finger and respond to the bending action of the finger in real-time based on the change of relative resistance. Moreover, it displays good sensitivity (GF = 2.05 at 100% strain) and working stability (stably cycling for 10 min). Additionally, the PVA/GO strain sensor can also be used as a flexible writing keyboard, accurately identifying the English letters written on it. This facile and mass-produced ion-conducting hydrogel can be further developed and applied for smart, flexible, and energy storage devices [40].

## 2. Materials and Methods

### 2.1. Materials

Polyvinyl alcohol (PVA) (M_w_ = 146,000–186,000, 99+% hydrolyzed; Sigma-Aldrich, St. Louis, MO, USA), crystalline flake graphite (99.9% metals basis; Aladdin, Shanghai, China), potassium nitrate (KNO_3_, AR, 99%; Innochem, Gwinnett County, GA, USA), sulfuric acid (H_2_SO_4_, ca. 96% solution in water; Acros, Shanghai, China), potassium permanganate (KMnO_4_, 99+%, ACS reagent; Acros, Shanghai, China), hydrogen peroxide (H_2_O_2_, AR, 30 wt% solution in water; Innochem, Shanghai, China), and sodium chloride (NaCl, AR, 99.5%; Innochem, Shanghai, China) were used as received. The used ultrapure water was prepared by the Millipore system (18.2 MΩ cm).

### 2.2. Preparation of GO

The preparation method of GO is based on the previous work [41]. Briefly, in the water bath agitator, we carefully added 1.5 g of crystalline flake graphite and 1.5 g of KNO_3_ to 70 mL of concentrated sulfuric acid. We then heated the mixture to 40 °C, slowly adding 9.0 g of KMnO_4_ and stirring at 400 rpm for 6 h. Then, 120 mL of deionized water was carefully added to the mixture, heated to 60 °C and stirred for 30 min. Whereafter, 300 mL of deionized water was added again. After reacting for 5 min, we slowly dropped a few drops of H_2_O_2_ to reduce the residual KMnO_4_ and MnO_2_ until the color of the reaction solution turned bright yellow. After repeatedly centrifuging the mixture with deionized water until it became neutral, the mixture was freeze-dried to obtain GO nanosheets.

### 2.3. Preparation of PVA, GO and Salt Solutions

PVA powder (10 g) was added to deionized water (90 g), and 10 wt% PVA solution was prepared under vigorous stirring at 95 °C. After cooling to room temperature, it was degassed by sonication for 30 min to obtain a clear PVA solution. GO powder (0.5 g) was added with deionized water (99.5 g) and dispersed for 5 min in an ultrasonic crusher to obtain 0.5 wt% GO solution. NaCl powder (117 g) was added to a 1000 mL volumetric flask, and then the salt was dissolved with deionized water to obtain a NaCl solution (2 M).

### 2.4. Fabrication of PVA/GO Hydrogels

A total of 5 g PVA solution (10 wt%) and 5 g GO solution (0.5 wt%) were stirred and mixed first. After ultrasonic degassing for 10 min, the mixture of PVA (5 wt%)/GO (0.25 wt%) was obtained. The mixture was poured into a mold, and the mold was frozen at −20 °C for 4 h. Then, the mold was immersed in NaCl solution (2 M) for 4 h, and thus the PVA/GO (0.25 wt%) hydrogel was prepared. In the same way, PVA/GO (0 wt%), PVA/GO (0.05 wt%), and PVA/GO (0.15 wt%) hydrogels were prepared by adjusting the content of the GO solution.

### 2.5. Preparation of PVA/GO Strain Sensor

The PVA/GO hydrogel was first designed using molds of specific sizes. It was then carefully attached to a specific position on a stretchable insulating layer (VHB 4905) with copper wires. Note here that the PVA/GO hydrogel must be in contact with the copper wires. Then, another layer of VHB was used to encapsulate to obtain a simple strain sensor.

### 2.6. Conductivity Measurements

The resistance values of the PVA/GO hydrogels were measured with an LCR meter (TH 2830). Simply, the resistance of PVA/GO hydrogels with different GO contents (Length × Width × Height = 2 cm × 1.5 cm × 1 cm) was tested using an LCR meter. The resistivity was then calculated by the following formula:

*ρ* = *RS*/*L*, (1)

where *R* is the resistance of the samples, and *S* and *L* represent the cross-sectional area and length of the samples, respectively. Therefore, the conductivity (*σ*) was calculated through the following formula:

*σ* = 1/*ρ*, (2)

### 2.7. Characterization

Transmission electron microscopy (TEM), high-resolution transmission electron microscopy (HRTEM), and selected area electron diffraction (SAED) were used to investigate the microstructures of the GO nanosheets (FEI Tecnai F20). Atomic Force Microscopy (AFM) imaging (Bruker Dimension ICON) in tapping mode was performed on a sample of GO (0.1 mg mL^−1^) on freshly cleaved mica at a resolution of 1024 × 1024 lines and at a scan rate of 0.5 Hz. The chemical bonds of GO were studied by employing X-ray photoelectron spectroscopy (XPS) on an Axis Ultra DLD Kratos AXIS SUPRA spectrometer. UV-Vis was utilized to investigate the absorption peak of GO. X-ray diffraction (XRD) was used to characterize the GO, PVA hydrogel, and PVA/GO hydrogel. The XRD patterns were recorded using a PANalytical-Empyrean X-ray diffractometer equipped with Cu K*α* radiation (*λ* = 1.54 Å) with scanning at a rate of 4° min^−1^. Raman (Horiba Scientific LabRAM HR Evolution with a 532 nm excitation wavelength) spectra of GO, PVA hydrogel, and PVA/GO hydrogels were collected, ranging from 400 to 2400 cm^−1^. An intelligent attenuated total reflection Fourier transform infrared spectrometer (ATR-FTIR, Thermo Fisher Nicolet Is5) was utilized to analyze and identify the functional groups of GO, PVA hydrogel, and PVA/GO hydrogel ranging from 4000 to 400 cm^−1^. Thermal gravimetric analysis (TGA, NETZSCH STA 409 PC) was applied to ascertain the organic–inorganic content of GO, PVA hydrogel, and PVA/GO hydrogel at a heating rate of 10 °C min^−1^ in air, from room temperature to 800 °C. The tensile stress–strain curves of the hydrogel samples were recorded on an electronic universal material testing machine (Instron 5967) at a deformation rate of 1 mm min^−1^. The compression tests were performed at a deformation rate of 1 mm min^−1^ at 25 °C. An LCR meter (TH 2830) operated by LabView software collected all relative resistance change signals of the samples.

## 3. Results and Discussion

### 3.1. Preparing Illustration of PVA/GO Hydrogel

**Figure 1:**
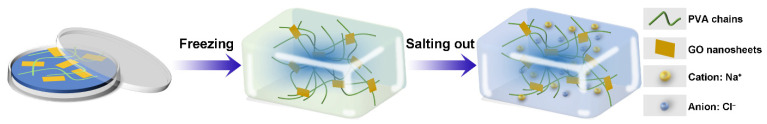
Schematic illustration of the formation process of PVA/GO ionic conductive hydrogel under the synergistic action of freezing and salting out.

PVA hydrogels have good mechanical strength and water retention capacity, as well as good biocompatibility and flexibility for artificial soft tissue applications [42]. Moreover, the polymer chains are entangled under the impact of high concentrations of salts, thereby enhancing the physical cross-linking of the polymer chains [43]. Meanwhile, NaCl has also been proven to impart high ionic conductivity to PVA hydrogels [33]. Therefore, we chose PVA to design and fabricate an ion-conducting hydrogel with a physically and chemically cross-linked network. Briefly, inspired by the Hofmeister effect [36,42,44], PVA and GO solutions in different proportions were rapidly mixed and poured into a mold (Figure 1). Subsequently, the molded PVA/GO solid ice cubes were obtained after placing the mold in a −20 °C refrigerator for 4 h. Next, a mechanically enhanced ion-conducting hydrogel PVA/GO was prepared after salting out the formed PVA/GO in a NaCl (2 M) solution at room temperature for 4 h. On the one hand, the salting-out process would make the PVA chains entangle to form a mechanically enhanced hydrogel. On the other hand, it would cause Na^+^ and Cl^−^ to evenly distribute inside the hydrogel and endow the hydrogel with excellent ionic conductivity. The porous network structure formed by the connection of sheets and PVA chains through hydrogen bonds and covalent bonds can further enhance the strength of the hydrogel and facilitate the transport of ions.

### 3.2. Characterization of GO and Preparation of PVA/GO Hydrogels

**Figure 2:**
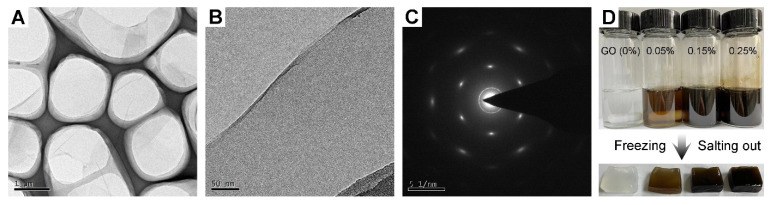
Morphology characterization of prepared GO. (**A**) Transmission electron microscope image of GO. (**B**) High-resolution transmission electron microscope images of GO. (**C**) The corresponding selected electron diffraction pattern. (**D**) Digital photos of PVA/GO ionic conductive hydrogels with different GO contents prepared under the synergistic effect of freezing and salting out.

Before preparing the PVA/GO hydrogel, the structure and composition of the as-prepared GO were firstly characterized to confirm that the prepared GO with the improved method was sufficiently exfoliated and oxidized. The transmission electron microscopy (TEM) images in Figure 2A showed that the as-prepared GO was a fully exfoliated thin layer. Further high-resolution transmission electron microscopy (HRTEM) images demonstrated that the as-prepared GO was a thin homogeneous layer (Figure 2B). The corresponding selected area electron diffraction (SAED) pattern in Figure 2C confirmed that the as-prepared thin-layer GO was fully oxidized. Therefore, PVA/GO solutions with GO contents of 0%, 0.05%, 0.15%, and 0.25% were prepared by controlling the content of GO added to the PVA solution. Subsequently, PVA/GO ion-conducting hydrogels incorporating different amounts of GO were fabricated with the synergistic process of freezing-salting out (Figure 2D).

**Figure 3:**
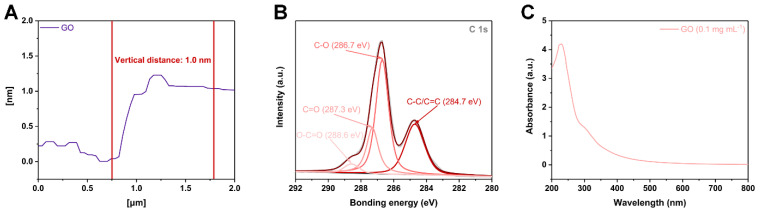
(**A**) Thickness of GO as measured by atomic force microscopy. (**B**) C1s High-resolution XPS spectra of GO. (**C**) UV-Vis spectrum of GO (0.1 mg mL^−1^).

Herein, atomic force microscopy (AFM) was used to measure the thickness of the GO (Figure 3A). The measured thickness of GO by AFM was about 1.0 nm, indicating that the applied GO had a single-layer structure. In addition, X-ray photoelectron spectroscopy (XPS) was utilized to characterize the chemical bonds of GO. The C1s spectrum of graphene oxide in Figure 3B manifested the existence of four carbon bonds: C–C/C=C (284.7 eV), CO (286.7 eV), C=O (287.3 eV), and O–C=O (288.6 eV), suggesting that GO was fully oxidized [45,46]. In addition, in Figure 3C, the ultraviolet-visible spectrophotometer (UV-Vis) results showed that GO (0.1 mg mL^−1^) had a main absorption peak at 230 nm and a shoulder peak at 300 nm, attributing to the *π*-π* transition of the C=C bond and the n-π* transition of the C=O bond, and also indicating that the applied GO was a homogeneous monolayer [45].

### 3.3. Characterization of PVA/GO Hydrogels

**Figure 4:**
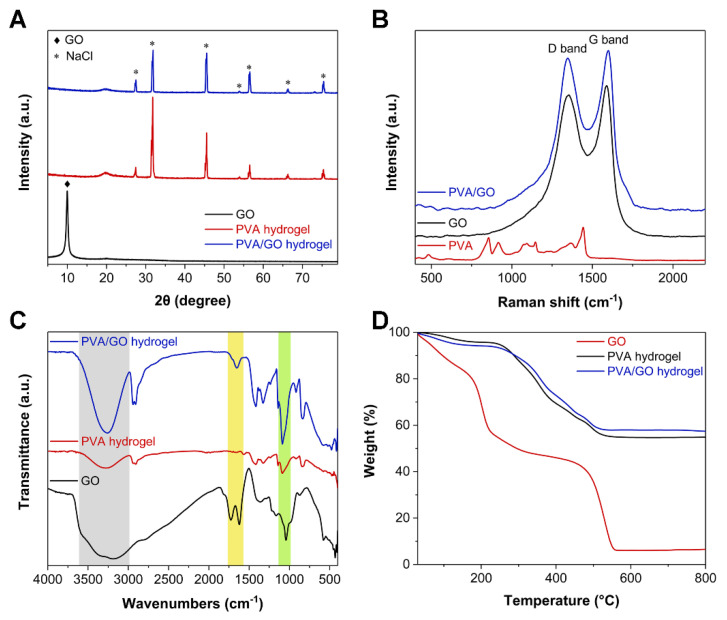
Chemical composition analysis of GO, PVA hydrogel, and PVA/GO hydrogel. (**A**) XRD patterns. (**B**) Raman spectra. (**C**) FTIR spectra. (**D**) TGA curves.

The color of PVA/GO hydrogel gradually deepened with the increase of GO content in the aforementioned Figure 2D. Thus, further characterization concerning GO nanosheets, pure PVA hydrogel, and PVA/GO (0.25 wt%) hydrogel was performed to determine how the addition of GO and the freezing–salting-out process impacted the formation of the hydrogel. The XRD pattern of GO in Figure 4A displays a characteristic peak around 10°, corresponding to the crystal plane (001) of GO. However, the XRD characteristic peaks of PVA hydrogel and PVA/GO hydrogel both displayed characteristic peaks of NaCl, which indicated that a large amount of NaCl entered and fixed in the interior of the hydrogel during the freezing–salting-out process. However, the characteristic peak of GO around 10° was not observed in the XRD pattern of the PVA/GO hydrogel because the interaction between PVA and GO led to the intercalation of PVA between the GO sheets. Further, Raman spectroscopy was used to characterize GO nanosheets, PVA hydrogels, and PVA/GO hydrogels. The Raman spectrum of the PVA/GO hydrogel in Figure 4B indicated the presence of the characteristic D and G bands of graphene, confirming the successful incorporation of GO into the hydrogel. Furthermore, the functional groups and chemical bonds of GO, PVA, and PVA/GO were analyzed using Fourier transform infrared spectroscopy (FTIR) (Figure 4C). The light green area was the characteristic peak of the hydroxyl group of PVA and PVA/GO hydrogels caused by C–O stretching vibration near 1186.7 cm^−1^. Additionally, the PVA/GO hydrogel peak was obviously enhanced here, indicating that PVA and GO were bound to each other. In the light-yellow area, the FTIR curve of PVA/GO at about 1654.1 cm^−1^ corresponded to the hydrogen bond formed between PVA and GO. In contrast, no peaks were observed for PVA hydrogels here. Furthermore, in the light-gray area, the intermolecular hydrogen bond (stretching vibration of the hydroxyl group) corresponding to the PVA/GO hydrogel was stronger than that of the PVA hydrogel near 3261.0 cm^−1^, indicating that there was an interaction between PVA and GO. Moreover, thermogravimetric analysis (TGA) was used to analyze the organic–inorganic content of GO, PVA hydrogels, and PVA/GO hydrogels (Figure 4D). After heating in air from room temperature to 800 °C, the PVA and PVA/GO hydrogels retained 54.6% and 56.8% of their mass, respectively, suggesting a large amount of ingress and immobilization occurred during the salting-out process. Na^+^ and Cl^−^ were inside the hydrogel, correlating to the XRD pattern results in Figure 4A. The PVA/GO hydrogel can adsorb more Na^+^ and Cl^−^ than the PVA hydrogel due to the more complex network structure formed between PVA and GO.

### 3.4. Tensile-Compressive Tests of PVA/GO Hydrogels

**Figure 5:**
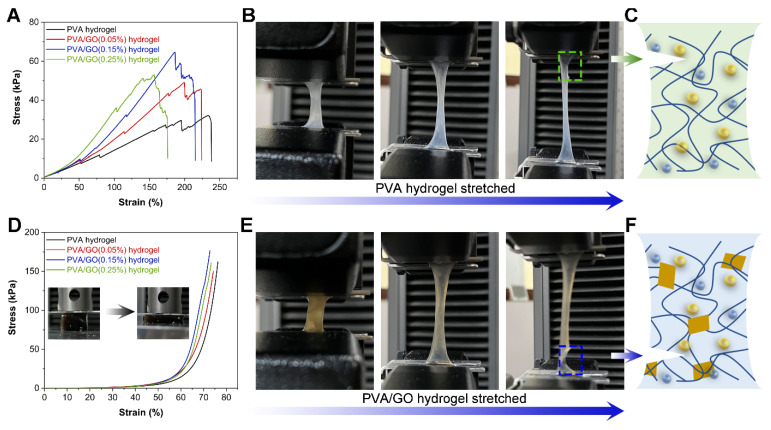
Mechanical properties of PVA/GO hydrogels with different GO contents. (**A**) Tensile stress–strain curves. (**B**) Tensile fracture process of PVA hydrogel. (**C**) Schematic diagram of ductile fracture mechanism of PVA hydrogel. (**D**) Compressive stress–strain curves. (**E**) Tensile fracture process of PVA/GO hydrogel. (**F**) Schematic diagram of enhanced ductile fracture mechanism of PVA/GO hydrogel.

The addition of GO led to forming a complex hydrogel network by PVA and GO. Henceforth, a universal electronic material testing machine was utilized to characterize the mechanical properties of PVA/GO hydrogels containing different amounts of GO. Hydrogels of a fixed PVA content (5 wt%), namely pure PVA, PVA/GO (0.05%), PVA/GO (0.15%), and PVA/GO (0.25%), prepared by the synergy of freezing–salting out in the mold, all exhibited excellent tensile properties in Figure 5A. With the addition of small amounts of GO, the hydrogels displayed a decreased maximum tensile length but a rising stress intensity. GO surfaces have a large number of hydrophilic functional groups, which can physically and chemically react with the PVA chain. Thereby the formed PVA complex network can exhibit enhanced mechanical properties. However, the excessive GO could not be cross-linked with PVA and would be stacked in the matrix network of PVA. Therefore, once the GO content increased to 0.25%, the PVA/GO hydrogels, on the contrary, displayed a decreased maximum tensile stress. In addition, a multi-step ductile fracture instead of one brittle fracture was observed from the tensile curves of the hydrogels. This phenomenon indicated that the prepared PVA/GO hydrogel exhibited excellent toughness. When the PVA hydrogel was gradually stretched to fracture, the fracture position occurred at both ends of the hydrogel, and the tear did not rapidly spread along the fracture (Figure 5B). This phenomenon happened because, after the salting out of the NaCl solution, the polymer chains of the PVA hydrogel were entangled with each other, thus preventing the propagation of cracks and yielding excellent toughness (Figure 5C). Similarly, if the fabricated PVA/GO hydrogel was gradually stretched to fracture, the fractures also occurred at both ends of the hydrogel (Figure 5E). However, after adding GO nanosheets, GO can further interact with the entangled PVA chains, thereby further enhancing the toughness of the hydrogel (Figure 5F). Comparable results can be observed in the compressive stress–strain curves of the hydrogels in Figure 5D. The strain strength of the hydrogel gradually increased as the GO content in the hydrogel increased from 0 to 0.15%. However, a further increase of the GO content to 0.25% contrarily yielded a decreased strain strength of the hydrogels.

### 3.5. Performances and Applications of the PVA/GO Strain Sensor

**Figure 6:**
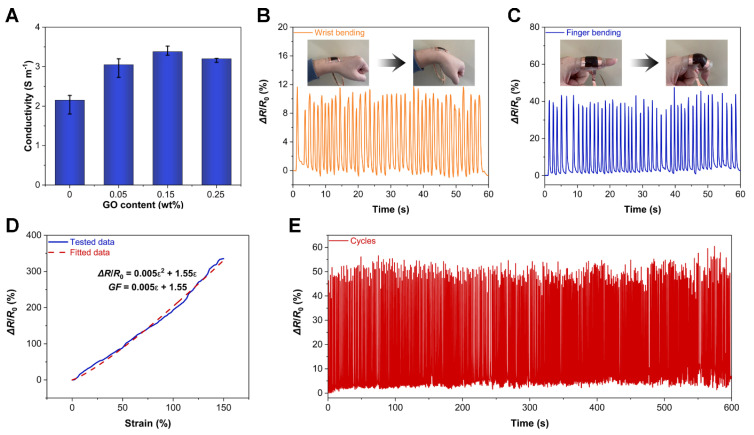
(**A**) Ionic conductivities of PVA/GO hydrogels with different GO contents. (**B**) The PVA/GO strain sensor responding to the bending motion of the wrist in real-time. (**C**) The PVA/GO strain sensor responding to the bending motion of the finger in real-time. (**D**) Sensitivity of the PVA/GO strain sensor. (**E**) Cyclic working stability of the PVA/GO strain sensor.

To further determine the enhancement of the ionic conductivity of the hydrogels by the salting-out process, the ionic conductivity of PVA/GO hydrogels with different GO contents was evaluated. The ionic conductivity of the PVA hydrogel after freezing–salting-out treatment reached 2.15 S m^−^^1^ (Figure 6A). When the GO content was 0.15%, the ionic conductivity of the PVA/GO hydrogel arrived at 3.38 S m^−^^1^. The measured enhancement of conductivity originated from the interaction between PVA and GO, yielding a formation of a complex network structure. These network structures can form channels, which are favorable for the transport of Na^+^ and Cl^−^, thus displaying better ionic conductivity. However, when the GO content was further increased to 0.25%, the electrical conductivity of the PVA/GO hydrogel, on the contrary, decreased. This decreased conductivity was due to the deteriorated transport efficiency of ions. Namely, the excessive GO could not be effectively cross-linked with the PVA chains and stacked in the PVA matrix network, which would consequently hinder the transport efficiency of Na^+^ and Cl^−^ within the hydrogel, thus manifesting a decrease in ionic conductivity.

Based on the previous analysis, the PVA/GO ion-conducting hydrogels with mechanical and ionic conductivity enhancements formed under the synergistic effect of freeze-salting precipitation are excellent candidates for applications in wearable devices and soft robotics. Therefore, a strain sensor can be fabricated simply by packaging the PVA/GO ion-conducting hydrogel onto a stretchable polyacrylate insulating tape to form a sandwich structure. When the PVA/GO strain sensor was attached to the wrist in Figure 6B, it could respond to the bending motion of the wrist in real-time with a relative resistance change (Δ*R*/*R*_0_) signal of about 10%. Likewise, in Figure 6C, the PVA/GO strain sensor can also monitor the bending motion of the finger in real-time with a Δ*R*/*R*_0_ of about 40%. Therefore, as shown in Figure 6D, by stretching the PVA/GO strain sensor to 150% strain, according to the previous methods [39,47], Δ*R*/*R*_0_ can be obtained by fitting the test data with the following formula:

Δ*R*/*R*_0_ = 0.005*ε*^2^ + 1.55*ε*, (3)

Therefore, the gauge factor (*GF*) followed the formula:

*GF* = 0.005*ε* + 1.55, (4)

when the strain was 100%, its *GF* arrived at 2.05. In addition, cyclic stability is also an important factor for the strain sensor. When the PVA/GO strain sensor was cycled for 10 min in Figure 6E, its working stability was still excellent as observed, revealing that the PVA/GO hydrogel after freezing-salting precipitation treatment had anti-fatigue properties.

**Figure 7:**
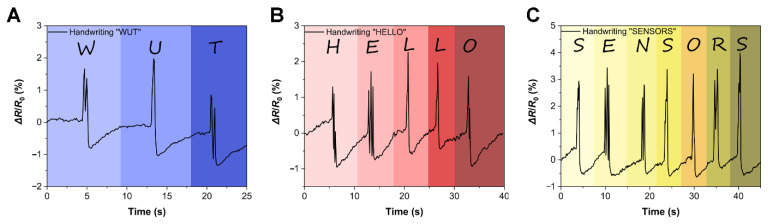
The PVA/GO flexible writing keyboard responding to the written English letters in real-time. (**A**) “WUT”. (**B**) “HELLO”. (**C**) “SENSORS”.

Furthermore, this PVA/GO strain sensor can also be used as a flexible writing keyboard. When writing “WUT” on the PVA/GO flexible keyboard in Figure 7A, it can respond in real-time with different Δ*R*/*R*_0_ signals, suggesting that this strain sensor demonstrated an accurate recognition function. Additionally, when writing “HELLO”, the PVA/GO flexible keyboard can also respond accurately in real-time (Figure 7B). In particular, the Δ*R*/*R*_0_ signal of the flexible keyboard exactly displayed the same responses when writing “L” twice. Thereby, this PVA/GO flexible keyboard can function with excellent stability. Moreover, we wrote “SENSORS” to further verify the practicality of the PVA/GO flexible keyboard in Figure 7C, confirming that the PVA/GO flexible keyboard can respond to stimuli in real-time, stably, and discriminately.

## 4. Conclusions

In this study, we demonstrated that introducing Na^+^ and Cl^−^ into the hydrogel can fabricate ionically conductive hydrogels of high toughness through the synergistic effect of freeze-salting during the preparation of PVA/GO hydrogels. The introduced GO can further react with the PVA chain to form a highly porous 3D network structure. This introduction can not only improve the mechanical strength of the hydrogel but also yield a higher ion transport efficiency of Na^+^ and Cl^−^ in the hydrogel network channel. Thereby the ionic conductivity of hydrogels can be further improved. Hydrogels are often difficult to be balanced with excellent mechanical strength and electrical conductivity. The PVA/GO ion-conducting hydrogels containing only 5 wt% PVA after salting out using NaCl and freezing exhibited strong toughness, high stress (65 kPa), and excellent electrical conductivity (up to 3.38 S m^−1^). Hydrogels with such properties are ideal candidates as strain sensors. The PVA/GO strain sensor was able to respond in real-time to the wrist and fingers’ bending motion and exhibited good sensitivity (GF = 2.05) at 100% strain. In addition, the PVA/GO strain sensor can also be used as a flexible writing keyboard, which can recognize the English letters written on it in real-time, stably, and accurately. This engineered, ionically conductive hydrogel has great potential for applications in wearable devices and soft robotics.

## Data Availability

All the data is available within the manuscript.
